# Multi-target tyrosine kinase inhibitor-associated renal thrombotic microangiopathy: a pooled analysis of 31 cases

**DOI:** 10.3389/fphar.2026.1834421

**Published:** 2026-04-22

**Authors:** Miao Liu, Xingchen Zhou

**Affiliations:** 1 Department of Pharmacy, Hunan Cancer Hospital/The Affiliated Cancer Hospital of Xiangya School of Medicine, Central South University, Changsha, Hunan, China; 2 Department of Pharmacy, Xiangya Hospital, Central South University, Changsha, Hunan, China

**Keywords:** adverse drug reaction, hypertension, proteinuria, renal injury, thrombotic microangiopathy, tyrosine kinase inhibitor

## Abstract

**Background:**

Multi-target tyrosine kinase inhibitors (TKIs) are cornerstone therapies for solid malignancies, yet their association with renal thrombotic microangiopathy (TMA)—a severe, irreversible adverse event—remains incompletely defined. This study delineates TKI-associated renal TMA’s clinical features, onset patterns, and outcomes.

**Methods:**

We systematically searched PubMed, Embase, Cochrane Library, and Web of Science for case reports/series of renal TMA linked to 7 multi-target TKIs (sunitinib, sorafenib, etc.) published through January 2026. Key data were extracted and analyzed descriptively.

**Results:**

Thirty-one cases were included (median age 62 years, 51.6% female). Common tumors: renal cell carcinoma (29.0%), thyroid cancer (16.1%), gastrointestinal stromal tumor (12.9%). Sunitinib was most implicated (48.4%). Median latency: 16 months (0.5–96 months); combination therapy shortened to 3.5 months. Dominant triad: hypertension (58.1%), AKI (77.4%), nephrotic proteinuria (67.9%); classic MAHA was uncommon. Kidney biopsy (87.1%) confirmed TMA. TKI discontinuation: 87.1% (6.5% dose reduction). Among evaluable patients, 90.0% improved, but 53.3% developed residual CKD and 10.0% ESRD.

**Conclusion:**

TKI-associated renal TMA presents as hypertension-proteinuria-AKI rather than classic hemolysis. Sunitinib confers highest risk, baseline hypertension is a key modifiable factor, and combination therapy accelerates onset. Lifelong monitoring and timely TKI discontinuation are critical, though residual CKD is common.

## 1 Introduction

Multi-target tyrosine kinase inhibitors (TKIs) have revolutionized the therapeutic landscape for a wide range of solid malignancies by selectively inhibiting key oncogenic and angiogenic signaling pathways, including the vascular endothelial growth factor receptors (VEGFRs) and platelet-derived growth factor receptors (PDGFRs) (Du et al., 2025; Shyam et al., 2023). Agents such as sunitinib, sorafenib, lenvatinib, and pazopanib have become standard-of-care therapies for advanced renal cell carcinoma, gastrointestinal stromal tumors, hepatocellular carcinoma, and differentiated thyroid cancer, and have significantly improved overall survival in patients with advanced-stage disease (Tomuleasa et al., 2024).

However, the anti-angiogenic mechanism that underpins the therapeutic efficacy of these agents also disrupts physiological vascular homeostasis, leading to a unique spectrum of adverse events (Sharma et al., 2021). The kidney, with its high-perfusion glomerular capillary network and heavy reliance on VEGF signaling for vascular integrity, is particularly susceptible to TKI-induced VEGF pathway inhibition (Leisring et al., 2024). Under physiological conditions, podocyte-derived constitutive VEGF is critical for maintaining glomerular endothelial fenestration, thromboresistance, and the integrity of the glomerular vascular barrier (Sharma et al., 2021; Leisring et al., 2024). Systemic VEGF signaling inhibition by TKIs thus induces direct glomerular endothelial injury, which may culminate in thrombotic microangiopathy (TMA)—a severe form of kidney injury pathologically defined by endothelial swelling, subendothelial space widening, glomerular basement membrane (GBM) double-contour formation, and intracapillary microthrombi (Largeau et al., 2023). Clinically, TKI-associated renal TMA presents with *de novo* or exacerbated hypertension, proteinuria (frequently in the nephrotic range), and progressive renal dysfunction, which may progress to irreversible ESRD if unrecognized and untreated in a timely manner (Leisring et al., 2024; van Doorn et al., 2025; Genest et al., 2023).

Despite accumulating case reports over 2 decades, comprehensive analyses of TKI-associated renal TMA—including its clinical phenotype, risk factors, and outcomes—remain lacking. Unanswered questions about incidence, predisposing factors, drug-specific risks, temporal patterns, and optimal management contribute to suboptimal vigilance, potentially delaying intervention and exposing patients to irreversible kidney damage.

To address this gap, we systematically analyzed published cases of multi-target TKI-associated renal TMA. Our objectives were to characterize patient profiles, delineate temporal onset patterns across TKIs and regimens, summarize clinico-pathological features, compare frequently implicated TKIs, evaluate interventions and outcomes, and identify instructive scenarios. By establishing a comprehensive clinical portrait, we aim to provide evidence-based insights to facilitate early recognition, accurate diagnosis, and optimal management of this severe adverse event.

## 2 Methods

### 2.1 Literature search and case identification

We systematically searched PubMed, Embase, Cochrane Library, and Web of Science from inception to January 2026 for case reports/series of renal TMA associated with multi-target TKIs (sunitinib, sorafenib, lenvatinib, pazopanib, regorafenib, cabozantinib, anlotinib) using keywords including “tyrosine kinase inhibitor”, “thrombotic microangiopathy”, and “renal injury”. Reference lists of relevant articles were manually screened. No language restrictions were applied.

Inclusion criteria (Du et al., 2025): treatment with multi-target TKIs (Shyam et al., 2023); development of renal TMA during/after therapy, defined by new/worsening hypertension, proteinuria, and/or AKI (Tomuleasa et al., 2024); TMA confirmed by kidney biopsy or by typical MAHA features (schistocytes, thrombocytopenia, elevated LDH, renal impairment) after excluding alternative etiologies.

Exclusion criteria (Du et al., 2025): TMA attributed solely to anti-VEGF monoclonal antibodies (e.g., bevacizumab monotherapy) (Shyam et al., 2023); renal injury from non-TKI agents or malignant progression (Tomuleasa et al., 2024); insufficient data (Sharma et al., 2021); duplicate publications.

Cases with TKI combinations (including bevacizumab or chemotherapy) were included and analyzed as a distinct “combination therapy” subgroup.

### 2.2 Data extraction

Two reviewers independently screened studies and extracted data using a standardized form, including: publication details; patient characteristics (age, sex, tumor type, baseline comorbidities); drug information (TKI, dose, latency, concomitant medications); clinical/laboratory findings (symptoms, Hb, platelets, LDH, SCr, proteinuria); diagnostic data (biopsy and histopathology); interventions (TKI discontinuation/reduction, PLEX, glucocorticoids, RAAS inhibitors, RRT); and outcomes (TMA resolution, renal recovery, re-challenge, survival). Laboratory values were standardized to international units; unavailable data were coded as “not reported”.

### 2.3 Statistical analysis

Descriptive statistics were used. Continuous variables are presented as medians (ranges) and categorical variables as frequencies (percentages). For incidence rates, denominators represent cases with available data. No meta-analysis or inferential tests were performed. Latency was analyzed separately for monotherapy and combination therapy groups, with stratification by individual TKI where sample size permitted.

## 3 Results

### 3.1 Patient and treatment characteristics

A total of 31 cases of multi-target TKI-associated renal TMA met the predefined inclusion criteria and were included in the final analysis (Wu et al., 2019; Singh et al., 2024; Ghimire et al., 2024; Bollée et al., 2009; Han et al., 2025; Costero et al., 2010; Jhaveri et al., 2011; Kapiteijn et al., 2007; Zhang et al., 2024; Khan et al., 2025; Talebi et al., 2012; Jha et al., 2013; Noronha et al., 2016; Levey et al., 2008; Yılmaz et al., 2016; Usui et al., 2014; Hanna et al., 2018; La Manna et al., 2018; Zhang et al., 2025; Delsante et al., 2022; Xue et al., 2024; Contreras et al., 2023; Nakashima et al., 2022; Yin et al., 2022; Kalla et al., 2021; Syed et al., 2018; Dai et al., 2024). Detailed case summaries are provided in the Supporting Information. Baseline characteristics are summarized in Table 1. Median age was 62 years (range 15–77), with female predominance (51.6%). Commonest tumors: renal cell carcinoma (29.0%), thyroid cancer (16.1%), and gastrointestinal stromal tumor (12.9%), together accounting for 58.1% of cases.

**TABLE 1:** Baseline characteristics of 31 patients with TKI-associated renal TMA.

Characteristic	Value (N = 31)
Age, years, median (range)	62 (15–77)
Sex, male/female, n (%)	Male 15 (48.4%)/Female 16 (51.6%)
Primary tumor type, n (%)	
Renal cell carcinoma	9 (29.0%)
Gastrointestinal stromal tumor	4 (12.9%)
Thyroid cancer	5 (16.1%)
Hepatocellular carcinoma	3 (9.7%)
Pancreatic neuroendocrine tumor	2 (6.5%)
Colorectal cancer	2 (6.5%)
Other tumor types*	6 (19.4%)
Implicated TKI, n (%)	
Sunitinib	15 (48.4%)
Lenvatinib	7 (22.6%)
Sorafenib	3 (9.7%)
Regorafenib	2 (6.5%)
Pazopanib	2 (6.5%)
Cabozantinib	1 (3.2%)
Anlotinib	1 (3.2%)
Treatment regimen, n (%)	
TKI monotherapy	25 (80.6%)
TKI combination therapy	6 (19.4%)
— With bevacizumab	3 (9.7%)
— With other agents^†^	3 (9.7%)
Key comorbidities, n (%)	
Baseline hypertension^‡^	11/30 (36.7%)
CKD/baseline renal insufficiency	5 (16.1%)

* Other tumor types include: malignant eccrine porocarcinoma (2 cases), transitional cell carcinoma, osteosarcoma, carcinoma of unknown primary, lung cancer, Von Hippel-Lindau syndrome.

† Other concomitant agents include: octreotide, gemcitabine, cyclosporine/immunosuppressants.

‡ Denominator is cases with available baseline data (1 case with unreported hypertension status).

Sunitinib was the most frequently implicated TKI, accounting for 15 cases (48.4%), followed by lenvatinib (7 cases, 22.6%) and sorafenib (3 cases, 9.7%). TKI monotherapy was the predominant treatment modality (80.6%), while combination therapy was administered in 6 cases (19.4%): 3 cases in combination with bevacizumab and 3 cases with other agents (octreotide, gemcitabine, or immunosuppressants). Among 30 evaluable patients with available baseline data, baseline hypertension was present in 36.7% (11/30), and pre-existing CKD or baseline renal insufficiency was documented in 16.1% (5/31) of the entire cohort.

### 3.2 Time from TKI initiation to TMA onset

The latency from TKI initiation to the clinical onset of renal TMA varied substantially across the study cohort (Table 2). Among 30 evaluable cases with available timing data, the overall median latency was 16 months (range, 0.5–96 months). For sunitinib monotherapy (n = 12), the median latency was 23 months (range, 0.5–96 months), with an exceptionally broad spectrum encompassing both hyper-acute onset (2 weeks) and ultra-chronic presentation (8 years). Lenvatinib monotherapy (n = 7) was associated with a median latency of 8 months (range, 1–28 months). For other TKIs administered as monotherapy (n = 5), the median latency was 23 months (range, 0.8–36 months), with significant inter-drug variability observed: pazopanib (0.8 months), sorafenib (4 months), regorafenib (23 months), anlotinib (29 months), and cabozantinib (36 months).

**TABLE 2:** Time from TKI initiation to renal TMA onset (N = 30*).

Treatment category (n)	Median time to onset (range)
All cases (N = 30)	16 months (0.5–96 months)
Sunitinib monotherapy (n = 12)	23 months (0.5–96 months)
Lenvatinib monotherapy (n = 7)	8 months (1–28 months)
Other TKI monotherapy (n = 5)^†^	23 months (0.8–36 months)
TKI combination therapy (n = 6)	3.5 months (1–26 months)
— With bevacizumab (n = 3)	6 months (5–26 months)
— With other agents (n = 3)^‡^	2 months (1–2 months)

* One case excluded due to unclear timing of TMA, onset relative to TKI, initiation.

† Other TKI, monotherapy includes: sorafenib (1 case, 4 months), regorafenib (23 months), pazopanib (0.8 months), cabozantinib (36 months), anlotinib (29 months).

‡ Other concomitant agents include: octreotide, gemcitabine, cyclosporine/immunosuppressants.

Notably, combination therapy was associated with a significantly shortened median latency of 3.5 months (range, 1–26 months) in all six patients who received this treatment regimen. Within the combination therapy group, the three patients treated with TKI plus bevacizumab had a median TMA onset of 6 months (range, 5–26 months), while the three patients receiving TKI in combination with other non-anti-VEGF agents (octreotide, gemcitabine, immunosuppressants) had an shorter median latency of 2 months (range, 1–2 months).

### 3.3 Clinical and laboratory manifestations

The clinical and laboratory features of TKI-associated renal TMA are summarized in Table 3. *De novo* or exacerbated hypertension occurred in 58.1% (18/31) of patients. Acute kidney injury (AKI) was documented in 77.4% (24/31), and nephrotic-range proteinuria (>3.5 g/24 h) was present in 67.9% (19/28) of evaluable patients. Peripheral edema was observed in 45.2% (14/31) of cases.

**TABLE 3:** Clinical and laboratory features of TKI-associated renal TMA (N = 31).

Feature category	Specific finding	Incidence (n/N)*	Notes
Clinical	New-onset or worsening HTN	18/31 (58.1%)	Most common; often severe or malignant
	Nephrotic-range proteinuria	19/28 (67.9%)	Core renal manifestation
	Acute kidney injury (AKI)	24/31 (77.4%)	Universal renal outcome
	Edema	14/31 (45.2%)	Related to hypoalbuminemia/sodium retention
Laboratory	Anemia (↓Hb)	17/22 (77.3%)	Common but not invariable
	Thrombocytopenia (↓PLT)	14/25 (56.0%)	Classic MAHA triad uncommon
	Elevated LDH	16/19 (84.2%)	Suggests microangiopathic hemolysis
	Low haptoglobin	8/10 (80.0%)	Suggestive of hemolysis; reported in 10 cases
	Schistocytes on smear	12/21 (57.1%)	Present in cases where peripheral smear was performed
	Low C3 complement	1/21 (4.8%)	Only one case with decreased serum C3; most cases had normal levels, consistent with direct endothelial injury mechanism

* Denominator is cases with available data for that specific variable. HTN, hypertension; Hb, hemoglobin; PLT, platelet; LDH, lactate dehydrogenase; SCr, serum creatinine; MAHA, microangiopathic hemolytic anemia.

In contrast, the classic MAHA triad was uncommon. Anemia (hemoglobin decline) occurred in 77.3% (17/22) of evaluable patients, thrombocytopenia in 56.0% (14/25), and elevated lactate dehydrogenase (LDH) in 84.2% (16/19). No patient presented with the complete classic MAHA triad, confirming that TKI-associated renal TMA is characterized by a kidney-predominant hypertension-proteinuria-renal injury pattern rather than florid systemic hemolysis.

In addition to the core laboratory parameters, we further analyzed haptoglobin, schistocytes, and complement C3 levels to provide a more comprehensive laboratory profile of TKI-associated renal TMA. Among 10 cases with available data, 8 (80.0%) had low haptoglobin, consistent with ongoing microangiopathic hemolysis. Peripheral smears were available in 21 cases, and schistocytes were detected in 12 (57.1%), a key diagnostic feature of TMA. Complement C3 levels were measured in 21 cases; only 1 (4.8%) showed decreased C3, while the remaining 20 were normal. This supports the view that complement activation is not a dominant mechanism in TKI-associated TMA.

### 3.4 Diagnostic approach and histopathology

Kidney biopsy played a pivotal role in the definitive diagnosis of TKI-associated renal TMA (Table 4). A total of 27 patients (87.1%) underwent a renal biopsy, and all biopsied cases demonstrated characteristic histopathological features of TMA. On light microscopy, the typical findings included GBM double contours (tram-track appearance), glomerular endothelial cell swelling, mesangiolysis, and intracapillary microthrombi. Electron microscopy further revealed subendothelial space widening, electron-dense deposition in the subendothelial region, and loss of glomerular endothelial fenestrations—pathognomonic features of TMA. The remaining 4 patients (12.9%) received a clinical diagnosis of TMA based on the presence of typical laboratory features of MAHA (schistocytes on peripheral blood smear, elevated LDH, thrombocytopenia) in the appropriate clinical context, after rigorous exclusion of alternative etiologies of renal injury.

**TABLE 4:** Diagnostic approach and kidney biopsy Findings (N = 31).

Diagnostic pathway	Cases, n (%)	Notes
Kidney biopsy-confirmed TMA	27/31 (87.1%)	Gold standard; confirmed TMA in all biopsied cases
Clinical diagnosis (no biopsy)	4/31 (12.9%)	Based on typical MAHA features after exclusion of other causes
Typical pathological findings	—	Light microscopy: GBM double contours, endothelial swelling, mesangiolysis, microthrombi. Electron microscopy: Subendothelial widening, electron-dense deposits, loss of fenestrations

GBM, glomerular basement membrane; MAHA, microangiopathic hemolytic anemia.

### 3.5 Comparative clinical features of different TKIs

A comparative analysis of the three most commonly implicated multi-target tyrosine kinase inhibitors (sunitinib, lenvatinib, and sorafenib) identified both shared clinical characteristics and distinct inter-agent differences (Table 5). Sunitinib accounted for the largest proportion of cases (48.4%), and all three agents were associated with high rates of *de novo* or worsening hypertension, underscoring the central role of VEGF signaling inhibition in the pathogenesis of TKI-associated renal TMA. Notably, sunitinib showed the widest latency range (0.5–96 months), whereas latency was substantially shorter for lenvatinib and sorafenib. All three drugs frequently induced severe nephrotic-range proteinuria, a sensitive clinical marker of glomerular endothelial injury in this setting. The spectrum of primary tumor types aligned with each drug’s approved indications: sunitinib was most commonly associated with renal cell carcinoma and gastrointestinal stromal tumor, lenvatinib with thyroid cancer, and sorafenib with hepatocellular carcinoma and renal cell carcinoma.

**TABLE 5:** Comparison of clinical features among major TKIs.

Feature	Sunitinib (n = 15)	Lenvatinib (n = 7)	Sorafenib (n = 3)	Clinical implication
Proportion of cases	48.4%	22.6%	9.7%	Sunitinib carries strongest risk signal
Median latency (mono)	23 months	8 months	4 months *	Significant inter-drug variability; sunitinib requires lifelong vigilance
Latency range (mono)	0.5–96 months	1–28 months	4 months *	Sunitinib exhibits widest latency spectrum
Associated hypertension	13/15 (86.7%)	7/7 (100%)	1/3 (33.3%)	Strict BP control is universal cornerstone
Proteinuria severity	Frequently nephrotic	Frequently nephrotic	Heavy proteinuria	Proteinuria is common sensitive marker

* Sorafenib monotherapy data based on single case; interpret with caution.

RCC, renal cell carcinoma; GIST, gastrointestinal stromal tumor; HCC, hepatocellular carcinoma; BP, blood pressure.

The small sample size for sorafenib (n = 3) and the combination therapy subgroup (n = 6) limits statistical power; findings for these subgroups should be interpreted cautiously.

Although sunitinib accounted for the largest number of cases, lenvatinib and sorafenib also carry non-negligible risks of renal TMA that should not be underestimated. Lenvatinib was associated with highly hypertension incidence and a shorter latency, indicating that its clinical toxicity should not be overlooked. In contrast, the true risk of sorafenib cannot be accurately assessed due to the extremely small sample size (n = 3, including only 1 monotherapy case), yet equivalent clinical vigilance is still required. The observed inter-drug differences are related to their pharmacological properties: sunitinib exerts potent dual inhibition against VEGFR and PDGFR-β, leading to the highest TMA risk; lenvatinib demonstrates stronger and more selective VEGFR inhibition, which may account for its earlier onset; sorafenib exhibits relatively mild anti-angiogenic activity, although no definitive conclusion can be drawn due to the limited sample size.

### 3.6 Treatment and renal outcomes

The therapeutic interventions and clinical outcomes of the study cohort are detailed in Table 6. Discontinuation of the offending TKI was the universal cornerstone of management, implemented in 87.1% (27/31) of all cases. In addition, two patients (6.5%) underwent dose reduction without complete discontinuation. Adjunctive therapeutic agents were used infrequently in this cohort: glucocorticoids in 16.1% (5/31) of cases and plasma exchange (PLEX) in 9.7% (3/31), primarily reserved for patients with severe systemic microangiopathic hemolytic anemia (MAHA) or central nervous system (CNS) involvement. Temporary renal replacement therapy (RRT) was required for acute renal failure in 6.5% (2/31) of patients, with all cases requiring only short-term renal support. Notably, the combination therapy group comprised only six cases, and the re-challenge subgroup comprised only two cases; these small sample sizes limit statistical power, and the findings should be interpreted with caution. Renin-angiotensin-aldosterone system (RAAS) inhibitors were widely used as supportive care in the majority of patients for the control of hypertension and proteinuria.

**TABLE 6:** Treatment interventions and clinical outcomes (N = 31).

Parameter	Finding	Key implication
Core intervention	TKI discontinuation: 27 (87.1%)	Cornerstone of management
Adjunctive therapy	Glucocorticoids: 5 (16.1%)	Used only in severe/systemic cases
	Plasma exchange (PLEX): 3 (9.7%)	Not routine; reserved for severe MAHA/CNS involvement
	RAAS inhibitors: Widespread	Essential supportive care for BP/proteinuria control
	Temporary RRT: 2 (6.5%)	Often transient
Final renal outcome*	Complete/near-complete recovery: 11 (36.7%)	Majority improve, but residual CKD very common
	Partial recovery (residual CKD): 16 (53.3%)	High burden of chronic kidney sequelae
	No recovery/ESRD: 3 (10.0%)	Real risk of permanent kidney failure
Patient survival	Death: 1 (3.2%)	Cause: tumor progression, unrelated to TMA
Time to TMA resolution	Median: 3 months (range: days-24 months)	Recovery can be slow
Subsequent cancer therapy	Switch to other class: common	Preferred strategy
	Successful re-challenge (reduced dose): 2 (6.5%)	High-risk option only in select cases

* Renal outcomes assessed in 30 patients with evaluable data (excluding one death from tumor progression).

CKD, chronic kidney disease; CNS, central nervous system; ESRD, end-stage renal disease; MAHA, microangiopathic hemolytic anemia; PLEX, plasma exchange; RAAS, renin-angiotensin-aldosterone system; RRT, renal replacement therapy; TMA, thrombotic microangiopathy.

The small sample size for the combination therapy group (n = 6) and the re-challenge subgroup (n = 2) limits statistical power; findings for these subgroups should be interpreted cautiously.

Among 30 patients with evaluable renal outcomes (excluding one death from tumor progression unrelated to TMA), the majority (90.0%, 27/30) demonstrated improvement in renal function after TKI discontinuation and supportive care. Specifically, 36.7% (11/30) achieved complete or near-complete recovery to baseline renal function, while 53.3% (16/30) developed residual chronic kidney disease (CKD). A small proportion (10.0%, 3/30) progressed to end-stage renal disease (ESRD) requiring permanent dialysis. Notably, the relatively high proportions of residual CKD and ESRD may be largely attributed to delayed diagnosis and prolonged TKI exposure, which lead to persistent glomerular endothelial injury, subsequent glomerulosclerosis and interstitial fibrosis. In most cases with unfavorable renal outcomes, clinicians failed to recognize progressive proteinuria and renal dysfunction in a timely manner, resulting in unnecessary continuation of TKI therapy for months. Correspondingly, renal biopsy in these patients typically demonstrated severe chronic pathological changes rather than reversible acute TMA lesions. The median time to clinical and laboratory resolution of TMA was 3 months (range, a few days to 24 months), with significant variability across the cohort. One patient (3.2%) died from tumor progression during the follow-up period, with death unrelated to TMA or renal dysfunction.

For subsequent anti-cancer therapy, the majority of patients were switched to agents with distinct mechanisms of action (e.g., mTOR inhibitors such as everolimus, immune checkpoint inhibitors such as nivolumab), representing the preferred strategy to avoid recurrent TMA. Only 2 patients (6.5%) underwent a successful TKI re-challenge with the same agent at a reduced dose under strict clinical monitoring after complete TMA resolution and renal function recovery.

### 3.7 Special clinical scenarios

Several clinically instructive scenarios emerged from the systematic review, which provide critical insights for the clinical management of TKI-associated renal TMA (Table 7). These cases highlight the diverse presentations, risk factors, and outcomes that clinicians should be aware of when managing patients on multi-target TKI therapy.

**TABLE 7:** Special clinical scenarios and key takeaways.

Scenario	Case example (Drug/Time/Key feature)	Clinical takeaway
Ultra-long latency	Sunitinib, onset at 8 years with nephrotic syndrome	Challenges “early risk only” dogma; mandates lifelong monitoring
Accelerated onset with combination therapy (anti-VEGF)	Sunitinib + bevacizumab, AKI at 5 months (Scr 2.3 mg/dL); Regorafenib + bevacizumab, massive proteinuria at 6 months (20.7 g/24 h); Sorafenib + bevacizumab, onset at 26 months	Combination anti-angiogenic therapy is a “risk multiplier”; requires ultra-vigilant monitoring from initiation
Accelerated onset with combination therapy (non-anti-VEGF)	Sunitinib + octreotide, onset at 1 month; Sorafenib + gemcitabine, onset at 2 months; Pazopanib + immunosuppressants, onset at 2 months	Combinations with non-angiogenesis inhibitors may also accelerate TMA; vigilance still required
Successful re-challenge	Sunitinib, resumed at 37.5 mg/day after complete recovery (initial proteinuria 6.80 g/24 h, resolved after 1 month)	High-risk option in desperate oncologic situations under strict monitoring
Delayed diagnosis → ESRD	Sunitinib, proteinuria ignored, TKI continued, ESRD at 32 months	Early recognition and prompt cessation are critical to prevent irreversible damage
Malignant hypertension with CNS involvement	Sunitinib, presented with seizures, vision loss, BP 210/110 mmHg, LDH 6505 U/L, platelets 26 × 10^9^/L	TMA can present as a hypertensive emergency with multi-organ involvement

AKI, acute kidney injury; CNS, central nervous system; ESRD, end-stage renal disease.

Some scenarios are based on limited cases, clinical application should consider individual circumstances.

The ultra-long latency case (sunitinib, TMA onset at 8 years) challenges the conventional clinical wisdom that TKI-associated toxicities occur only in the early phase of treatment and underscores the necessity of lifelong renal monitoring. Accelerated onset with combination therapy (sunitinib + bevacizumab at 5 months; regorafenib + bevacizumab at 6 months; sorafenib + bevacizumab at 26 months) highlights the amplified risk of combined anti-angiogenic regimens, which act as a risk multiplier for renal TMA. A successful TKI re-challenge case (sunitinib resumed at 37.5 mg/day after complete TMA resolution) provides a potential—though high-risk—treatment option for patients with no alternative anti-cancer therapies. Conversely, a case of delayed TMA diagnosis leading to ESRD (proteinuria ignored, sunitinib continued for 32 months) tragically underscores the critical importance of early recognition and prompt TKI discontinuation to prevent irreversible renal damage. Malignant hypertension with CNS involvement (sunitinib presenting with seizures and vision loss) reminds clinicians that TKI-associated renal TMA can present as a hypertensive emergency with multi-organ involvement, requiring emergent management. Notably, TKI combinations with non-anti-angiogenic agents (sunitinib + octreotide at 1 month; sorafenib + gemcitabine at 2 months; pazopanib + immunosuppressants at 2 months) also appeared to accelerate TMA onset, warranting heightened clinical vigilance even with these non-anti-angiogenic combinations.

## 4 Discussion

This systematic study of 31 rigorously selected cases provides the first comprehensive clinical portrait of multi-target TKI-associated renal TMA, quantifying its key features, risk patterns, temporal kinetics, and prognostic trajectories to offer evidence-based guidance for clinical management.

First, this study identifies sunitinib as the TKI with the strongest risk signal for renal TMA and confirms baseline hypertension as a core patient susceptibility factor. Sunitinib-related cases accounted for 48.4% of the cohort, suggesting a unique pharmacological risk profile. Mechanistically, sunitinib’s dual inhibition of VEGFR and PDGFR-β is the likely underlying cause of its heightened risk (Jin et al., 2023). VEGF signaling ablation directly impairs glomerular endothelial cell survival and barrier function, leading to loss of endothelial fenestrations and downregulation of anticoagulant factors (Andres-Jensen et al., 2022; Giri et al., 2024; Finch et al., 2022; Martin Alonso et al., 2026). Concurrently, PDGFR-β inhibition weakens pericyte support, rendering endothelial cells more vulnerable to shear stress. The synergistic action of these mechanisms results in severe endothelial dysfunction and TMA (van Doorn et al., 2025; Cui et al., 2022; Zhang et al., 2023; Aw et al., 2025). Notably, 36.7% of patients had pre-existing hypertension, which increases endothelial shear stress and oxidative stress (Du et al., 2025), creating a “double hit” that lowers the threshold for renal TMA. Baseline hypertension is a well-recognized independent risk factor for anti-angiogenic therapy-related renal adverse events (Kuang et al., 2024; Narayan et al., 2023), underscoring the need for optimal blood pressure control before TKI initiation.

Second, our analysis elucidates the unique temporal kinetics of TKI-associated renal TMA. The median latency of 16 months (range 0.5–96 months), including a case with onset after 8 years, refutes the notion that TKI toxicities are confined to early treatment. This extraordinarily wide spectrum—from acute to ultra-chronic—mandates lifelong renal monitoring, even in patients who have tolerated therapy for years. The conventional model of “early intensified monitoring followed by gradual relaxation” is therefore inadequate for preventing this complication.

Even more concerning, TKI combination therapy significantly shortened latency to TMA onset. The combination group (n = 6) had a median latency of only 3.5 months, versus 8–23 months for monotherapy. Within this group, TKI plus bevacizumab had a median onset of 6 months, while TKI with other non-anti-VEGF agents (octreotide, gemcitabine) had an even shorter latency of 2 months, suggesting synergistic amplification of renal toxicity. Mechanistically, combining TKIs with anti-VEGF antibodies achieves near-complete VEGF inhibition, enhancing anti-tumor efficacy but also increasing endothelial injury risk (Leisring et al., 2024; Java et al., 2025). For patients receiving combination regimens, ultra-stringent monitoring—including weekly blood pressure and monthly renal function tests—should be implemented from treatment initiation.

This study also reveals that the clinical manifestations of TKI-associated renal TMA are highly atypical, explaining its frequent underdiagnosis. The majority of patients presented with a hypertension-proteinuria-AKI triad, while classic MAHA was uncommon (<40%). This aligns with previous reports that only 28% of VEGF inhibitor-related renal injury cases exhibit typical MAHA (1). TKI-induced renal TMA represents a kidney-predominant microangiopathy, with injury concentrated in VEGF-dependent glomerular endothelium; systemic hemolysis appears only in late stages (Maisons et al., 2024). This explains why most patients in our cohort were diagnosed in the progressive stage, before advancing to systemic MAHA. When confronted with elevated SCr in TKI-treated patients, clinicians often attribute it to volume depletion, nephrotoxic medications, or tumor progression, excluding TMA from the differential diagnosis. Such delays may extend for months, leading to unnecessary treatment adjustments and potentially irreversible renal injury. Therefore, *de novo* or uncontrollable hypertension accompanied by rapidly progressive proteinuria or a >30% SCr increase should immediately prompt TMA investigation.

The laboratory findings further support the diagnosis of TMA and help clarify its pathogenesis. Hypohaptoglobinemia was observed in 80.0% of patients, consistent with ongoing microangiopathic hemolysis. Schistocytes, a hallmark of intravascular hemolysis, were present in 57.1% of patients with evaluable smears, confirming the presence of mechanical hemolysis. Notably, complement C3 levels were normal in 95.2% of cases (20/21), with only one case showing mild reduction. This pattern strongly supports the current understanding that TKI-associated TMA is driven primarily by direct endothelial toxicity, not complement overactivation (Genest et al., 2023). In contrast, complement-mediated TMA (e.g., atypical HUS) is typically accompanied by overt hypocomplementemia (Che et al., 2024). The minimal complement activation in our cohort suggests that anti-complement therapies are unlikely to be routinely warranted in most patients with TKI-induced TMA. These results are consistent with prior studies reporting absent complement deposition in TKI-associated renal TMA, further validating a non-complement-mediated pathological mechanism.

Renal biopsy plays a central and irreplaceable role in the diagnosis, prognostic stratification, and clinical management of TKI-associated renal TMA. In our cohort, more than three-quarters of patients (87.1%) were definitively diagnosed by kidney biopsy, with all specimens demonstrating classic histopathological features of TMA, including GBM double contours, endothelial swelling, mesangiolysis, intracapillary microthrombi, and subendothelial widening. These highly specific findings represent the diagnostic gold standard and provide critical guidance for clinical decision-making, especially regarding whether to discontinue potentially effective anticancer therapy. Without pathological confirmation, clinicians face two major pitfalls: either failure to withdraw the culprit TKI in reversible TMA, leading to irreversible renal damage, or unnecessary discontinuation of effective treatment due to non-TMA etiologies, resulting in missed therapeutic opportunities.

Notably, renal biopsy also enables semiquantitative evaluation of disease chronicity and provides essential prognostic information. Patients with delayed diagnosis and prolonged TKI exposure developed extensive glomerulosclerosis and interstitial fibrosis on biopsy, corresponding to irreversible renal injury that ultimately progressed to permanent dialysis despite drug cessation. In contrast, patients undergoing early biopsy with prompt TKI discontinuation predominantly exhibited active TMA lesions with minimal chronic changes and achieved substantial renal recovery. The degree of chronic pathological change is therefore a key determinant of irreversible renal damage and long-term renal prognosis (Drapeau et al., 2026; Gai et al., 2025). By distinguishing reversible active injury from irreversible chronic lesions, early renal biopsy directly informs critical management strategies, including TKI discontinuation, dose adjustment, and selection of subsequent anticancer treatments (van Doorn et al., 2025; Genest et al., 2023).

Regarding treatment and prognosis, timely TKI discontinuation was implemented in 87.1% of cases (plus 6.5% dose reduction). Among 30 evaluable patients, renal outcomes followed a tripartite pattern: 36.7% achieved complete/near-complete recovery, 53.3% developed residual CKD, and 10.0% progressed to ESRD. These outcomes convey two key messages: TMA is often reversible—90.0% of patients showed renal improvement, supporting active intervention—yet even with timely discontinuation, over half develop CKD, suggesting that TKI-induced endothelial injury may trigger irreversible fibrosis. Thus, early recognition and decisive TKI cessation are critical to prevent permanent renal failure, but long-term renal protection and cardiovascular risk management remain essential for all affected patients.

PLEX and glucocorticoids were used infrequently in our cohort (9.7% and 16.1%, respectively), and only in life-threatening cases (CNS involvement or severe MAHA). This practice aligns with the pathophysiology of TKI-associated renal TMA: direct endothelial toxicity is the primary mechanism, rather than complement abnormalities or autoimmune dysregulation (Largeau et al., 2023). A 2023 review confirms that PLEX has limited efficacy in direct drug toxicity-driven TMA, including TKI-associated cases (Mazzierli et al., 2022). Glucocorticoids are similarly ineffective in endothelial damage-mediated TMA and may delay definitive management (Mazzierli et al., 2022). Our data support this: among four PLEX recipients, only two achieved complete recovery, showing no clear advantage over TKI discontinuation alone.

For subsequent anti-cancer therapy, most patients were switched to agents with distinct mechanisms (e.g., mTOR inhibitors, immune checkpoint inhibitors), the preferred strategy to minimize TMA recurrence. Only two patients (6.5%) underwent successful dose-reduced TKI re-challenge after complete recovery, a potential option for those with high tumor progression risk and no alternatives. However, re-challenge is a high-risk decision requiring multidisciplinary consensus, fully informed consent, and strict monitoring (weekly blood pressure, bi-weekly renal function). At the first sign of TMA recurrence, the TKI must be permanently discontinued.

Based on our findings, we propose a structured clinical management pathway for optimizing renal safety in patients receiving multi-target TKI therapy. This pathway comprises five key components.

Pre-treatment risk assessment serves as the first line of defense. Before initiating TKI therapy, a systematic evaluation of the patient’s baseline clinical status should be conducted. This includes ensuring blood pressure control with a target of <130/80 mmHg, optimizing antihypertensive therapy if needed, and assessing baseline renal function (eGFR) along with quantitative proteinuria via 24-h urinary protein excretion or the urine protein-to-creatinine ratio. It is also essential to identify high-risk patient populations, such as those with baseline hypertension, a solitary kidney (e.g., post-nephrectomy), pre-existing mild renal insufficiency (eGFR <60 mL/min/1.73 m^2^), or those scheduled to receive combination anti-angiogenic therapy. For these high-risk individuals, the frequency of in-treatment monitoring should be intensified, and prophylactic use of RAAS inhibitors should be considered to mitigate vascular endothelial injury.

Stratified in-treatment monitoring is the core clinical strategy for achieving early recognition of TKI-associated renal TMA. Based on the latency data from our study, we recommend the following schedule. For all patients receiving TKI monotherapy, blood pressure, urinalysis, and serum creatinine should be monitored monthly for the first 3 months after treatment initiation, and then every two to 3 months indefinitely. For high-risk patients (those on combination therapy, with baseline hypertension, or with pre-existing CKD), monitoring should be performed monthly for the first 6 months, followed by every one to 2 months thereafter. For long-term TKI users (more than 2 years), clinical vigilance must not be relaxed due to prolonged treatment tolerance; regular monitoring every three to 6 months should be maintained, with particular attention to gradually progressive trends in proteinuria or serum creatinine.

Diagnostic triggers act as the critical bridge connecting routine monitoring to timely clinical action. Immediate nephrology consultation should be initiated upon the occurrence of any of the following clinical or laboratory findings: *de novo* hypertension or pre-existing hypertension that becomes difficult to control (requiring two or more antihypertensive agents for adequate control); the emergence of proteinuria from a baseline of none, or an increase from microalbuminuria to more than 1 g/24 h (or a urine protein-to-creatinine ratio greater than 1); a more than 30% increase in serum creatinine from baseline, or a more than 25% decline in eGFR; or unexplained anemia, thrombocytopenia, or elevated lactate dehydrogenase.

The diagnostic pathway emphasizes the core and irreplaceable value of kidney biopsy for pathological diagnosis. After rigorous exclusion of other clear causes of renal injury (such as volume depletion, urinary tract obstruction, or other nephrotoxic medications), kidney biopsy is strongly recommended to establish a definitive diagnosis of TMA. Biopsy not only confirms the presence of TMA but also assesses the chronicity of renal injury by distinguishing active lesions from chronic fibrotic changes. This assessment directly influences the aggressiveness of subsequent treatment decisions. For TMA predominantly characterized by active lesions, decisive TKI discontinuation offers the potential for complete renal function reversal. For those with extensive chronic changes, treatment decisions need to weigh more heavily the requirements for tumor control and patient survival.

Definitive treatment principles guide the implementation of specific clinical interventions for TKI-associated renal TMA. Upon high clinical suspicion or definitive pathological diagnosis of TMA, the causative TKI should be immediately discontinued, unless the need for tumor control is extremely urgent and TMA manifestations are mild, in which case short-term clinical observation with daily monitoring may be considered after multidisciplinary team discussion. RAAS inhibitors (such as irbesartan or lisinopril) should be actively used to control blood pressure and reduce proteinuria, with a target blood pressure of <130/80 mmHg. Short-term glucocorticoids or plasma exchange should be considered only for patients with severe systemic MAHA (hemoglobin <8 g/dL, platelets <50 × 10^9^/L, significantly elevated LDH) or central nervous system involvement. If indicated, temporary renal replacement therapy should be provided for acute renal failure. Subsequent anti-cancer treatment decisions require formulation by a multidisciplinary oncology-nephrology team, with priority given to switching to non-VEGF pathway targeted drugs (such as mTOR inhibitors or immune checkpoint inhibitors).

### 4.1 TKI rechallenge

For patients at extremely high risk of tumor progression with no alternative therapies, TKI rechallenge may be considered only when the following conditions are met: extremely high risk of tumor progression with no other effective alternatives; the initial TMA episode was mild (e.g., only mild proteinuria with no acute kidney injury); renal function has completely returned to baseline after TKI discontinuation; the patient provides fully informed consent and accepts stringent long-term monitoring; and the rechallenge dose is reduced by at least 25%–50% with concomitant RAAS inhibitor protection. Based on our two successful cases and current guidelines, we further propose detailed inclusion and exclusion criteria, a standardized monitoring protocol, and clear discontinuation thresholds to enhance clinical applicability.

Inclusion criteria require complete TMA remission with blood pressure <130/80 mmHg, stable renal function (eGFR ≥60 mL/min/1.73 m^2^ for at least 4 weeks), significant proteinuria improvement without nephrotic-range relapse, normalized complement C3/C4 levels, higher risk of tumor progression than TMA recurrence, and signed informed consent with monitoring compliance. Exclusion criteria comprise severe irreversible renal injury (eGFR <30 mL/min/1.73 m^2^) or diffuse glomerulosclerosis on biopsy, prior early TMA recurrence within 2 weeks after rechallenge, uncontrolled severe cardiovascular comorbidities, and biopsy-proven complement-mediated TMA. A standardized monitoring protocol is recommended, with weekly assessments in the first 4 weeks and every 4 weeks thereafter for at least 6 months, covering serum creatinine, eGFR, proteinuria, blood pressure, complement C3/C4, complete blood count, and electrolytes. Discontinuation thresholds include a ≥30% increase in serum creatinine, UACR ≥1.0 g/g, uncontrolled hypertension, or new-onset hemolytic markers. Based on our cases and current guidelines, we further propose a stepwise clinical pathway involving thorough pre-rechallenge evaluation, initial dosing at 50%–75% of the original dose or switching to a milder TKI, rigorous dynamic monitoring, and prompt treatment adjustment. The two successful cases in this study met all inclusion criteria and complied with the monitoring protocol without TMA recurrence. While real-world studies support clinical benefits in carefully selected patients, larger prospective investigations are still needed to validate this pathway and optimize individualized decision-making for TKI rechallenge.

### 4.2 Study limitations

This study has several limitations. First, as a systematic review of case reports, it is inherently subject to publication bias, and thus the true incidence of TKI-associated renal TMA cannot be estimated. Second, incomplete data reporting in some cases has led to smaller denominators for certain variables (e.g., lactate dehydrogenase (LDH) was recorded in only 19 cases), which may reduce the precision of the analysis. Third, variable and inconsistent follow-up durations have precluded the reliable assessment of long-term renal prognosis. Fourth, the overall small sample size (n = 31) and particularly the very limited size of the combination therapy subgroup (n = 6) significantly restrict statistical power, preventing robust comparative or subgroup analyses; therefore, all results, especially those from subgroup comparisons, should be interpreted with extreme caution. Finally, the findings may not be fully generalizable to other TKI agents or broader patient populations. It should be emphasized that this study is an original research independently carried out based on clinical data, in which all data, research design, and analysis conclusions are independently completed by the research team, without relying on existing reviews, secondary data, or other external materials, fully reflecting the originality and independence of the research.

### 4.3 Future research directions

Future research should focus on several key directions. First, a prospective, multicenter registry specifically for patients receiving multi-target TKIs should be established to systematically collect baseline characteristics, real-time monitoring data, pathological findings, treatment interventions, and long-term renal outcomes. This design will enable more accurate estimation of the real-world incidence of TKI-associated renal TMA and identification of definitive risk factors, thereby generating high-quality evidence to support clinical practice. Exploring the potential role of complement activation in multi-target TKI-associated TMA and the feasibility of anti-complement therapy in these patients represents an important avenue for future investigation. This will require prospective studies to collect complement-related laboratory parameters and conduct dedicated clinical trials. Second, early predictive biomarkers (e.g., urinary VEGF, complement activation products) should be explored to identify high-risk patients before renal function decline. Third, interventional studies evaluating prophylactic RAAS inhibitors are warranted. Fourth, emerging evidence suggests that TKI-induced gut dysbiosis may trigger metabolic reprogramming of gut microbiota, leading to endotoxemia or accumulation of uremic toxins (e.g., trimethylamine N-oxide, TMAO) and subsequent systemic inflammation, which exacerbates endothelial injury and potentially promotes TMA pathogenesis Citation (Wang et al., 2026). As highlighted by Wang et al. (2026), targeted modulation of gut microbiota (e.g., supplementation with Akkermansia muciniphila or *Lactobacillus* spp.) or fecal microbiota transplantation may serve as promising adjuvant strategies, opening new avenues for reducing TMA risk or improving renal outcomes by restoring gut-renal crosstalk. Additionally, pharmacogenomic studies may clarify the genetic basis for differential TKI risk, enabling personalized treatment strategies.

## 5 Conclusion

Multi-target TKI-associated renal TMA is a severe and distinctive adverse event of anti-angiogenic therapy, characterized by a hypertension-proteinuria-renal injury triad instead of classic systemic hemolytic anemia. Our systematic study of 31 cases confirms that sunitinib poses the strongest risk for this complication, baseline hypertension is a key susceptibility factor, and combination therapy markedly shortens the latency to TMA onset. Given the extremely wide latency window of up to 8 years, lifelong renal vigilance is mandatory for all patients receiving long-term TKI treatment. Kidney biopsy is essential for the definitive diagnosis and prognostic assessment of TKI-associated renal TMA, while timely discontinuation of the offending TKI is the cornerstone of clinical management. Although most patients achieve renal function improvement after TKI cessation, residual chronic kidney disease remains a common sequela, and a small proportion progress to irreversible end-stage renal disease. Clinicians should maintain a high index of suspicion for this atypical complication and implement structured surveillance strategies for early recognition and intervention. These findings establish a comprehensive clinical framework for managing TKI-associated renal TMA, which is critical to balancing effective cancer control and the preservation of long-term renal health in patients with malignancies.

## Data Availability

The original contributions presented in the study are included in the article/supplementary material, further inquiries can be directed to the corresponding author.
